# Assessing the Effects of Kata and Kumite Techniques on Physical Performance in Elite Karatekas

**DOI:** 10.3390/s20113186

**Published:** 2020-06-03

**Authors:** Luca Molinaro, Juri Taborri, Massimo Montecchiani, Stefano Rossi

**Affiliations:** 1 Department of Economics, Engineering, Society and Business Organization (DEIM), University of Tuscia, 01100 Viterbo, Italy; luca.molinaro@unitus.it (L.M.); stefano.rossi@unitus.it (S.R.); 2 Motustech – Sport & Health Technology c/o Marilab, 00121 Ostia Lido, Rome, Italy; 3 FIAMME ORO – Polizia di Stato, 00148 Rome, Italy; m.montecchiani@gmail.com; 4 FIJLKAM – Italian Federation of Judo, Wrestling, Karate and Martial Arts, 00100 Rome, Italy

**Keywords:** karate, sport biomechanics, inertial sensors, body stability, joint mobility, jumping

## Abstract

This study aimed at assessing physical performance of elite karatekas and non-karatekas. More specifically, effects of kumite and kata technique on joint mobility, body stability, and jumping ability were assessed by enrolling twenty-four karatekas and by comparing the results with 18 non-karatekas healthy subjects. Sensor system was composed by a single inertial sensor and optical bars. Karatekas are generally characterized by better motor performance with respect non-karatekas, considering all the examined factors, i.e., mobility, stability, and jumping. In addition, the two techniques lead to a differentiation in joint mobility; in particular, kumite athletes are characterized by a greater shoulder extension and, in general, by a greater value of preferred velocity to perform joint movements. Conversely, kata athletes are characterized by a greater mobility of the ankle joint. By focusing on jumping skills, kata technique leads to an increase of the concentric phase when performing squat jump. Finally, kata athletes showed better stability in closed eyes condition. The outcomes reported here can be useful for optimizing coaching programs for both beginners and karatekas based on the specific selected technique.

## 1. Introduction

Karate is a popular Japanese martial art with over 10 million athletes and 100 million practitioners in the world [1]. The popularity and scientific interest of karate grew up in the last decades, when the World Karate Federation has been recognized by the International Olympic Committee, and it will make its first appearance as an Olympic Sport at the 2020 Summer Games in Tokyo. Karate competition consists in a sequence of attacks and defenses by using punches and kicks with high speed and power [2]. Karate is basically divided into two main techniques that represent the two disciplines in the world tournaments: kumite and kata [3]. The word kumite means sparring; thus, karatekas use the basic techniques in interfering conditions during the competition against an adversary. Kumite matches last 3 min, and a high intensity activity, concerning kicks, punches, and quick horizontal displacements, is required [4]. It is easily understandable that the main aspects that should be trained in kumite karatekas are perceptual and anticipatory skills [5]. Conversely, kata competition consists in a standardized sequence of offensive and defensive gestures without the presence of the opponent; thus, it can be considered as a virtual fighting [6]. Both competitions are characterized by periods of lower intensity and periods of maximum work [7]; thus, maximal speed and explosive power are crucial elements of karate performance [8]. In the past, the coaching programs were similar for both techniques, and most of the athletes successfully participated in both kumite and kata during official competitions. However, the revised rules of the competitions made kumite more dynamic than kata; consequently, a proper specialization should be required in elite karatekas based on the specific technique [9].

The technological innovations of the last years allowed using wearable sensor systems for motor performance evaluation [10,11], also in combat sports [12,13,14]. In order to select the best discipline between kata and kumite for each karateka, information regarding physical and physiological characteristics of the athletes have to be taken into account [15]. More specifically, physiological aspects include cardiorespiratory endurance, muscular strength, and body composition; instead, physical characteristics include speed, stability, power, mobility, coordination, and agility [16]. 

In literature, several studies have been already conducted on the physiological and physical differences between kata and kumite. Those studies were mainly focused on the evaluation of metabolic consumption [17], stability [1,18], agility [4,19,20], mobility, and power [20]. 

As regards metabolic consumption, Doria et al. found a greater request of metabolic power in kumite players rather than kata ones (in average 155.8 mL/kg vs. 87.8 mL/kg), with a predominance of the aerobic contribution [17]. By focusing on the stability skills, Gauchard and colleagues evaluated the postural performance in elite kumite and kata comparing them with sedentary subjects when performing static balance control in different visual and tactile conditions, i.e., open and closed eyes and on different tactile supports. As expected, sedentary subjects showed a greater body sway than the athletes when performing bipodalic tasks; in addition, focusing on the two disciplines, kata players were characterized by a reduction of the body and area sway in both visual and tactile conditions [1]. Specifically, kata showed a reduction from 40 to 100 mm^2^ considering all the tested conditions. Authors suggested that such findings can be related to the different postural strategies needed for the specialization of the karatekas. Mirmoezzi et al. found that kumite athletes showed a better dynamic balance, even though this improvement decreased with fatigue caused by intense training [18]. Based on these findings, authors suggested to implement training programs to delay fatigue effects especially in kumite karatekas. Moving to the agility evaluation, Syaquro et al. did not find any differences associated with the reaction time between the two disciplines during whole-body rotation; conversely, they found a difference in the reaction speed (in average kumite = 0.32 s and kata = 0.23 s). In particular, kumite showed best results ascribable to the necessity of kumite to rapidly react to the opponent movements during the competition [19]. In the same context, Nedeljkovic et al. evaluated the reaction time in both offensive and defensive actions performed by kumite, kata, and beginners. Authors found differences only between beginners and others, while a promising but not significant difference was observed between kumite and kata, with the first one characterized by lower values of the reaction time [4]. In addition, the study performed by Zemkova revealed that the agility index was significantly better in kumite players than kata ones (on average 270 ms vs. 330 ms); the opposite result was obtained for the movement velocity [21]. As regards the mobility and power, only Koropanovski et al. aimed at identifying the differences due to the kumite and kata disciplines on anthropometric characteristics and physical performance [20]. Authors implemented a protocol involving different tasks to evaluate mobility of lower limbs, agility, power, and endurance, finding that an explosive power could be fundamental in kumite technique, whereas a smaller stature and a higher mobility of the lower extremity could be relevant for kata competitors. Authors suggested as the reported findings could be useful for an early selection of karate competitors.

To the best of authors’ knowledges, no studies have been carried out to investigate the differences due to the karate techniques on the upper limb mobility, jumping skills, and monopodalic stability in elite karatekas. Nevertheless, the mobility of upper limbs is one of the basic fitness components in karate for the execution of full-range movements at high speeds [15]. In addition, jumps and monopodalic stability represent motion tasks often required during competitions [22]. From this perspective, the study aims were twofold. We wanted, firstly, to assess the differences in motor performance between elite karatekas and non-karatekas, aiming at creating reference values of physical performance in elite karatekas useful to address beginners towards the most appropriate training programs. Secondly, we aimed at measuring the influence of karate specializations on physical performance in elite athletes; in particular, we wanted to assess the differences between kata and kumite players in the terms of whole-body joint mobility, body stability, and skills in jumping. The outcomes of the second aim could be useful to provide guidelines for the optimization of the coaching programs based on the specific karate technique.

## 2. Materials and Methods

### 2.1. Participants

Twenty-four international level karatekas (11 men and 13 women) with at least 15 years of practice and belonging the Italian national karate team were involved in the study. More specifically, 16 of them were kumite athletes (KU—seven males and nine females, age = 24 ± 6 years, height = 169.4 ± 7.6 cm, body mass = 64.8 ± 9.4 kg) and the remaining eight kata athletes (KA—four males and four females, age = 26 ± 5 years, height = 165.4 ± 5.6 cm, body mass = 66.8 ± 4.4 kg). Among them, there were winners of the Italian championship, European champions, and World champions in different weight categories. All of them were involved in karate six days per week and twice for each day. In addition, 18 age-matched subjects (thirteen men and five women, age = 25 ± 6 years, height = 171.2 ± 8.6 cm, body mass = 69.8 ± 10.4 kg) were involved in the study as non-karatekas (NK). Subjects were included in the NK group if they did never practice karate and they practice physical activities at maximum twice per week at a noncompetitive level. Participants, both karatekas and non-karatekas, had not any recent injuries for at least 2 years. All participants were informed on the rationale of the study, and a written informed consent was obtained according to the ethical standards outlined in the 1964 Declaration of Helsinki.

### 2.2. Experimental Setup

Two sensor systems were used in the experimental protocol: the OPTOGait and the GyKo. 

The OPTOGait system (OPTOGait, Microgate S.r.I, Italy, 2010) consists in a set of transmitting-receiving 1 m long bars positioned above the ground. Each bar is equipped with ninety-six LED diodes. Data were recorded using OPTOGait Version 1.12.1.0 software (Microgate S.r.I, Italy). Through this device, it is possible to record flight and contact times of each foot with an accuracy of 1 ms. The sampling frequency was set at 1 kHz. The OPTOGait system is commonly used in the analysis of human movements, especially for gait [23,24] and jump gesture analyses [25].

The GyKo (Microgate S.r.I, Italy) contains the MPU9250 that is a single Inertial Measurement Unit (IMU) (dimensions: 50 × 70 × 20 mm, mass: 35 g) consisting in a triaxial accelerometer (full scale ranged from ±2 g to ±16 g), a triaxial gyroscope (full scale ±2000 °/s) and a triaxial magnetometer (full scale ±4800 µT). The device can be fixed on a body segment using a dedicated semi-elastic belt equipped with a device-specific magnetic support to avoid relative movements during the execution of the tasks. Elastic belt guarantees also the subject comfort. Data gathered from the sensor is transferred via Bluetooth to a personal computer and stored using the GyKoRepower software version 1.1.2.0 (Microgate S.r.I, Italy). The sampling frequency was set at 500 Hz. The MPU9250 is characterized by an accuracy of ±0.1° in the angle computation [26]. The GyKo inertial sensor is mainly used to evaluate motor activities in jump tests [27], muscle strength test [28], and stability tests [29]; and its measures have been demonstrated to be reliable in both angle [30] and postural parameters computation [31]. 

Finally, two Webcams (Logitech C920 HD) were set at 3 m from the subject and were aligned with her/his frontal and lateral planes. Video were synchronized with data acquisition in order to record movements of the subject during the entire protocol. Videos were successively analyzed to verify the correctness of the task execution in order to evaluate tasks to discard from the analyses.

### 2.3. Experimental Protocol

The experimental protocol consisted of three tasks for the evaluation of whole-body joint mobility, monopodalic stability, and jumping ability. All the physical exercises were developed according to the requirements of the technicians of the Italian national karate team. All tests were carried out directly on the mat in barefoot condition. Before each task, a static acquisition with the sensorized body segment in rest position was performed. The rest position coincided with the starting position specific for each individual test, as described in the following paragraphs. All participants were able to complete the entire protocol, which lasted approximatively 45 min per subject.

#### 2.3.1. Joint Mobility

As regards joint mobility tasks, participants were asked to perform selected movements of shoulder, hip, and ankle joints with the greatest possible excursion at the preferred velocity without making compensations with other parts of the body. In addition, only for the shoulder movements and hip flexion movement, participants were asked to move body segments also at the maximum velocity (MV). Each required movement was repeated one time. Participants were instrumented with the GyKo placed on the body segment proximal to the examined joint in order to measure the linear acceleration and angular velocity of the segment, which the sensor was attached on. The full scale of the accelerometer embedded into the GyKo was set at ±2 g in mobility tasks. The OPTOGait sensor was not used in the mobility task.

##### Shoulder

**Figure 1:**
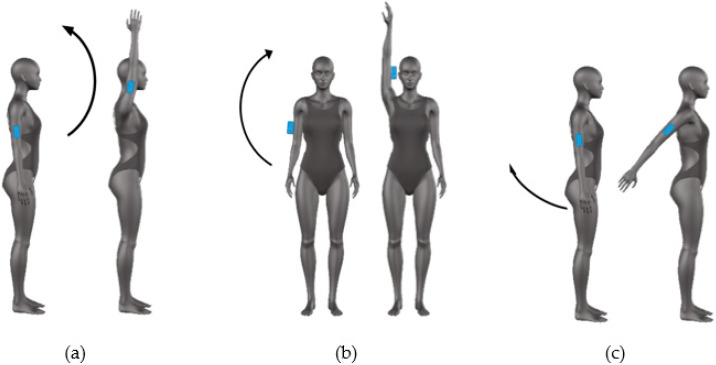
Mobility of shoulder: sensor positioning and movement direction for the flexion (**a**), abduction (**b**), and extension (**c**) task.

**Figure 2:**
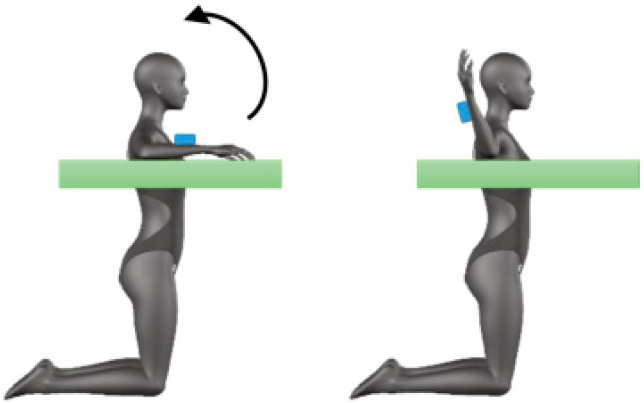
Mobility of shoulder: sensor positioning and movement direction for the extra-rotation task.

For the analysis of shoulder mobility, four movements were performed: flexion, extension, abduction, and extra rotation. As regards the first three movements, participants were asked to stand on the mat and to perform the requested rotations starting with the arms along the body (Figure 1); for the extra rotation, instead, subjects started with an abduction of shoulder equal to 90° resting the forearm and the hand on a flat support, as it is shown in Figure 2. During flexion and extension movements, subjects were requested to avoid intra and extra rotations; instead, natural physiological movements were allowed during the abduction rotation. The device was placed on the subject’s arm at 15 cm from the center of rotation of the shoulder during the flexion, extension, and abduction movements (Figure 1a,b). For the extra-rotation movement, the sensor was placed on the forearm at 15 cm from the elbow (Figure 1c) All the movements were performed with both sides and at both preferred and maximum velocity. Each movement was repeated one time. 

##### Hip

**Figure 3:**
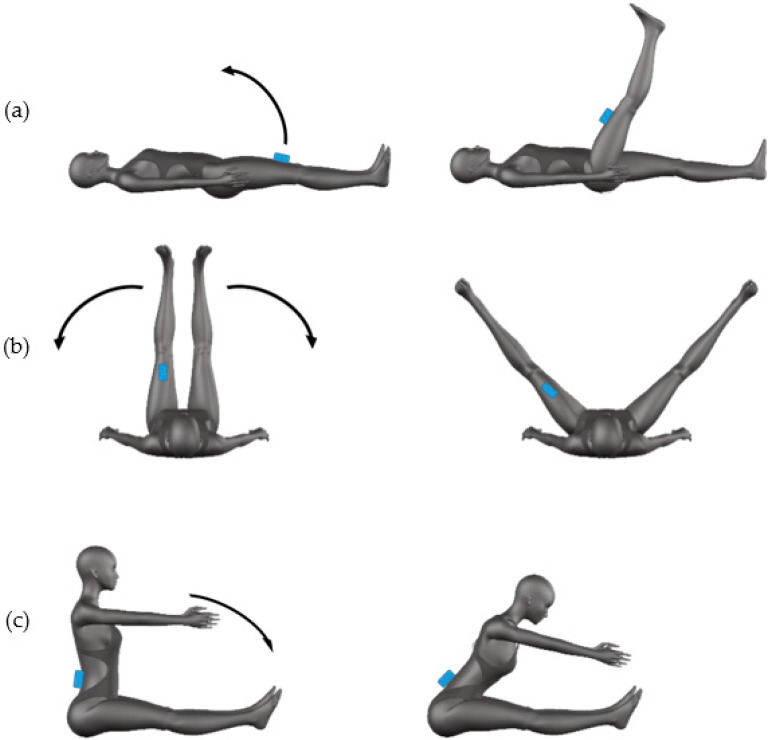
Mobility of hip: positioning of the sensor and movement direction for flexion (**a**), split (**b**), and sit and reach (**c**) task.

For the analysis of the hip mobility, three movements were performed. The first movement was a hip flexion starting from the supine position with the legs resting on the ground (Figure 3a); subjects were asked to avoid knee bending. The second movement, denominated split movement, consisted in a hip abduction performed with the two limbs simultaneously, starting with legs resting on the wall and the trunk extended on the ground; the hip flexion was maintained at 90° during the rotation (Figure 3b). Finally, the third movement, denominated sit and reach, consisted in a further hip flexion performed with the trunk and starting from the sitting position with the legs straight on the floor (Figure 3c); the rotations simulated a reaching movement [32]. The sensor was placed on the thigh at 15 cm from the knee center of rotation during the flexion and split movements (Figure 3a,b). For the sit and reach movement, the sensor was placed on the back at level L4 (Figure 3c). The flexion movement was performed with both sides; the split movement was repeated two times in order to measure one movement per each leg. Only the flexion movement was performed at both preferred and maximum velocity. 

##### Ankle

**Figure 4:**
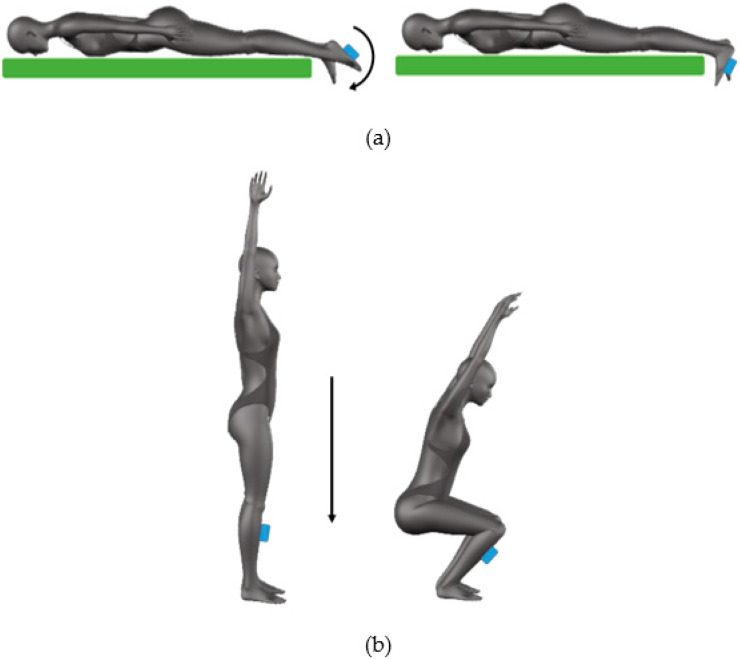
Mobility of ankle: positioning of the sensor and movement direction for flexion (**a**) and squat (**b**) task.

For the analysis of the ankle mobility, two movements were performed. The first movement was an ankle flexion caused by the foot rotation starting from a fully extended position of foot with the subject lying prone on the couch and the feet outside of it (Figure 4a). The second movement was an ankle flexion caused by the leg rotation during an overhead squat (Figure 4b). The sensor was positioned on the plantar fascia of the foot (Figure 4a) during the flexion task and on the shank at a distance of 10 cm below the knee during the squat movement (Figure 4b). The flexion movement was performed with both sides; the squat movement was repeated two times in order to measure movement one time per each leg. Ankle movements were performed only at the preferred velocity.

#### 2.3.2. Body Stability

As concerns body stability task, a novel monopodalic test was developed starting from the One-Leg Standing Balance [33,34] and modifying the position of the arms and the raised leg in accordance with the indications provided by the karate technicians. More specifically, participants were asked to stand on one foot with hip, knee, and ankle of the other leg flexed at 90° for 15 s, maintaining the hands crossed behind the head.

**Figure 5:**
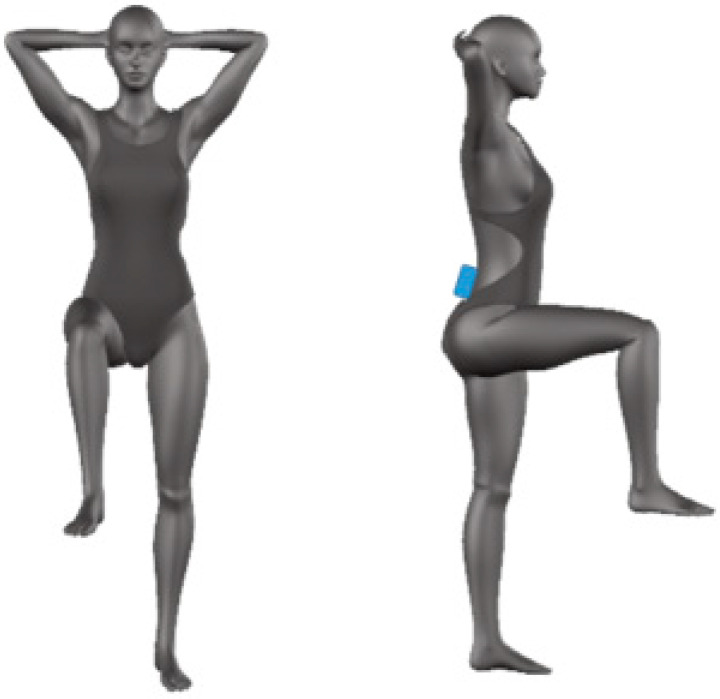
Balance test: positioning of the subject and sensor.

Participants were instrumented with the GyKo placed on the back at level L4 in order to measure the linear acceleration and angular velocity of the pelvis, which the sensor was attached on (Figure 5). For each subject, the distance between the sensors and the ground was measured. The full scale of the accelerometer embedded into the GyKo was set at ±2 g in stability tasks. The OPTOGait sensor was not used in the balance task. Test was performed with the dominant leg and with both open and closed eyes (EO and EC). During the EO condition, subjects were asked to fix a point on the wall at 5 m. The test was considered completed if the subject did not touch the ground with the raised limb or if he/she opened the eyes in EC condition within 15 s. Each condition was recorded one time after an initial period needed to familiarize with the task. 

#### 2.3.3. Jumping Ability

**Figure 6:**
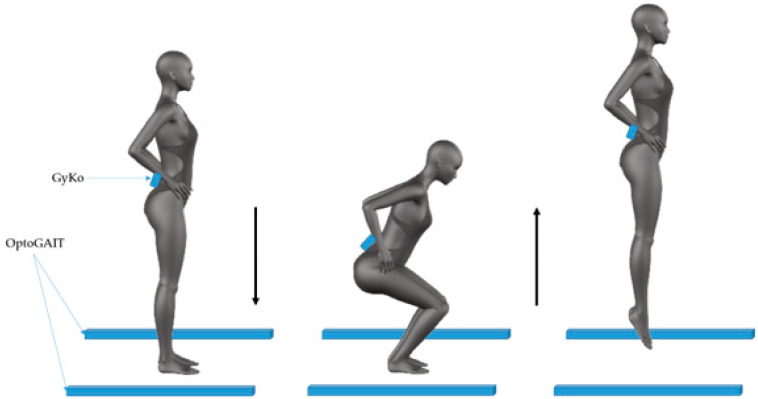
Jump test: subject and sensor position and sequence of movements.

As regards the analysis of jumping motor performance, jump tests included Squat Jumps (SJ), Countermovement Jumps (CMJ), and Repeated Countermovement Jumps for 15 s (RCMJ) [20,35]. In both jumps, the subjects were asked to start from a standing position with their feet shoulder-width apart and their hands on the iliac crests. In SJ, the athletes descended into a semi-squat position and held this position as long as they wanted before initiating the successive upward/concentric phase to jump. Conversely, in the CMJ the subjects performed a downward movement, which is immediately followed by the concentric phase without the maintaining of the semi-squat position [36]. All the jumps were carried out on the mat, without the help of the upper limbs, i.e., subject were instructed to maintain the hands attached to the body for the whole task. Participants took a 2-min rest period between each test to avoid fatigue effects [37,38]. Participants were asked to jump as high as possible without any restrictions of the knee angle during the squat phase. SJ and CMJ were repeated two times, instead the RCMJ was performed only one time since it allowed to evaluate the fatigue effects. Participants were instrumented with the GyKo placed on the back at L4 level to collect the linear acceleration and angular velocity of the pelvis. The full scale of the accelerometer embedded into the GyKo was set to ±16 g in jumping tasks. The OPTOGait system was positioned on the tatami with bars at 2.5 m (Figure 6).

### 2.4. Data Analysis

Data gathered during static trial was used for the re-alignment of the sensor axes with the absolute reference system; successively linear accelerations and angular velocities gathered from the GyKo were processed using a sensor fusion algorithm and the Mahony filter to compute the orientation of the sensor [39]. This post-processing operation was conducted for all the performed tests. Videos recorded during the task execution were carefully checked to discard trials in the following cases: (i) incorrect movements during the mobility tests; (ii) inability to perform the monopodalic tests, i.e., touch the ground with contralateral leg or open the eyes during the EC condition; and, (iii) incorrect position and execution during jumping task. Only the second situation occurred, as reported in the Section 3.2.

#### 2.4.1. Joint Mobility

As regard mobility tests performed at the preferred velocity, we defined as θ the highest difference between the angle computed during the execution of the movement and the one gathered at the start of the task with the body segment in the rest position, along the examined axis. Successively, $\dot{\theta }$ was defined as the average velocity of the performed movement.

By moving to the tests performed also at the maximum possible velocity (MV), i.e., all shoulder movements and the hip flexion, the same values were computed, and they were addressed as $\theta_{MV}$ and ${\dot{\theta }}_{MV}$.

#### 2.4.2. Body Stability

Body stability was analyzed considering the projection of the vertical axis of sensor on the horizontal plane corresponded to the floor. The point represented by this projection was evaluated knowing the distance between the position of GyKo sensor and the floor that was initially assessed by a measuring tape. The evaluated point can be assumed as the center of pressure (COP); then, the antero-posterior (AP) and the medio-lateral components (ML) of the COP were computed. Finally, as stability indices we computed parameters that are typical considered in posturographic analysis [40,41,42]. Specifically, the path length (PL), the ellipse area (EA) and the mean frequency (FREQ) were evaluated by following the equations reported in Reference [41]. 

The path length (PL) was defined as the total length of the COP path computed by the sum of the distances between two consecutive points of the COP path (Equation (1)).

(1) $$\mathrm{PL}=\sum_{n=1}^{N-1}{\left[{\left(\mathrm{AP}\left[n+1\right]-\mathrm{AP}\left[n\right]\right)}^{2}+{\left(\mathrm{ML}\left[n+1\right]-\mathrm{ML}\left[n\right]\right)}^{2}\right]}^{1/2},$$

where N represents the number of points of the COP. The PL was also evaluated considering the antero-posterior (PL_AP_) and medio-lateral (PL_ML_) components one at a time. 

The 95% confidence ellipse area (EA) was computed representing the area of the 95% bivariate confidence ellipse, which was expected to enclose approximately 95% of the COP (Equation (2)).

(2) $$\mathrm{EA}=2{\pi F}_{0.05\left[2,N-2\right]}{\left[s_{\mathrm{AP}}^{2}s_{\mathrm{ML}}^{2}-s_{\mathrm{APML}}^{2}\right]}^{1/2},$$

where $F_{0.05\left[2,N-2\right]}$ is the *F* statistic at a 95% confidence level for a bivariate distribution with *N* points. $s_{\mathrm{AP}}^{2}$ and $s_{\mathrm{ML}}^{2}$ are the variance of the AP and ML, and $s_{\mathrm{APML}}^{2}$ is the covariance.

The mean frequency (FREQ) was defined as the angular frequency of the COP if it had traveled the total excursions around a circle with a radius equal to the mean distance (MD), and it was evaluated as (Equation (3)):

(3) $$\mathrm{FREQ}=\frac{\mathrm{VEL}}{2\pi \mathrm{MD}}$$

where VEL was the average velocity of the COP, and MD was the average distance of each COP point from the origin defined as the projection of the gravity vector on the floor (Equation (4)):

(4) $$\mathrm{MD}=\frac{1}{N}\sum_{n=1}^{N-1}\sum {\left[\mathrm{AP}{\left[n\right]}^{2}+\mathrm{ML}{\left[n\right]}^{2}\right]}^{\frac{1}{2}}\;n=1,\ldots ,N,$$

and VEL was the average velocity of the COP. The FREQ parameter was also evaluated considering the antero-posterior (FREQ_AP_) and medio-lateral (FREQ_ML_) components individually.

The stability indices were calculated by eliminating the first 3 s of the test to discard the transition phase where the subject moved from the relaxed position, on two feet, to the monopodalic position. 

For all the above-mentioned parameters, the Romberg Index (RI) was also computed as the ratio between the stability parameters obtained during the EC and EO conditions [40]. Values of RI close to 1 indicate a better adaptation to the closed eyes condition. 

#### 2.4.3. Jumping Ability

As concerning the jump tests, the time instants in which the subject hit and detached himself from the ground were estimated by using the OPTOGait system. The linear acceleration gathered from the GyKo was integrated to obtain the linear velocity in order to identify the eccentric and concentric phases of the jumps. Specifically, the eccentric phase is identified as the time interval between the start of downward movement and the instant of time in which the subject reverses the movement, i.e., the zero of the velocity, and starts pushing upwards. The concentric phase, instead, is defined as the time interval between the end of the eccentric phase and the instant in which the subject detaches himself from the ground. In the SJ, the Flight Time (T_F_) and the Concentric Phase Time (T_CP_) were evaluated. In the CMJ, we also computed the Eccentric Phase Time (T_EP_). For both SJ and CMJ, all indices were averaged across the two repetitions for each participant. All previous parameters were computed also during RCMJ, and, in addition, we evaluated the Contact Time (T_C_).

### 2.5. Statistical Analysis

Data computed for all the examined tasks was firstly tested for normality with the Shapiro-Wilk test. As regard, the index computed for the mobility test, a one-way ANOVA test was performed considering the three examine groups as independent variables and by considering one index per time. Thus, a total of 28 ANOVA tests were performed considering each mobility index independently. Similar steps of statistical analysis were performed for each index related to the jumping test for a total of 9 ANOVA tests. As regards the body stability task, one-way ANOVA test was performed considering the three examined groups as between-subject effect. It is worth highlighting that the results obtained for the two visual conditions were tested independently. Thus, a total of 21 ANOVA tests were performed considering each stability index independently. For all the ANOVA tests, a Bonferroni Post-Hoc multiple comparison test was used to determine differences among means when ANOVA test was significant. Statistical significance was set at *p* < 0.05 for all the performed tests. The statistical power analysis was performed through the G*Power software [43]. Power values ranged from 82% to 91% were found, considering a medium effect size (0.5).

## 3. Results

### 3.1. Joint Mobility

Mean values and standard errors of the angles computed during the joint mobility tasks performed at the preferred velocity, and the relative statistical results are reported in Table 1. Right and left values were considered together due to similar results between the two sides obtained in a preliminary analysis within each group.

Regarding the θ value for shoulder mobility, differences in all movements were found between the KU and NK groups (*p* always lower than 0.01), with the KU group showing the highest values for all movements. The only difference between KU and KA was found for the extension movement (*p* = 0.03), where the KU group showed a greater value. In the same movement no difference was observed between KA and NK (*p* = 0.45). Considering movement velocity, KU group was always associated with a statistically greater value of $\dot{\theta }$ than the other groups (*p* always lower than 0.01). In addition, KA showed the lowest value for all the movements also with respect to the NK. It can be observed that the values of the standard error of $\dot{\theta }$ were always greater when focusing on karatekas with respect to NK. 

By moving to the hip mobility, all the values of θ were statistically different between the NK and the karatekas (*p* always lower than 0.01); in these cases, the only significant difference between KU and KA was observed in the flexion movement (*p* = 0.02), where KU showed greater values. The $\dot{\theta }$ parameter was always statistically different between KU and NK (*p* always lower than 0.01), whereas, between KU and KA, the differences were found for all movement (*p* ranged from 0.01 to 0.04) with the exception of the Sit and Reach (*p* = 0.70). KA and NK showed no different behavior only for the flexion movement (*p* = 0.12). 

As regards the movements related to the ankle, values of θ obtained during the flexion movement showed significant differences among all groups (*p* ranged from <0.01 to 0.04); instead, no difference between KU and NK was found for the overhead squat (*p* = 0.59). Generally, the KA group showed greater values for both movements. By moving to the velocity of the movements, no differences were found (*p* ranged from 0.07 to 0.53). 

Mean values and standard errors of the angles computed during the joint mobility task performed at the maximum speed, and the relative statistical results are reported in Table 2. Right and left values were considered together due to similar results between the two sides obtained in a preliminary analysis within each group.

Regarding the shoulder movement, karatekas showed $\theta_{MV}$ values always greater values than NK (*p* always lower than 0.01), but no difference was shown between KU and KA (*p* ranged from 0.43 to 0.88). In addition, the $\dot{\theta }$_MV_ parameter in all movements was statistically different between NK and the two groups KU and KA (*p* always lower than 0.01), while the only difference between KU and KA was observed in the extra-rotation movement (*p* = 0.01), with KU associated to the highest average values.

By moving to the hip, statistical lower value of $\theta_{MV}$ was found related to the NK (*p* < 0.01); instead, no difference was found between the two karatekas groups (*p* = 0.66). Similar outcomes were obtained for the $\dot{\theta }$_MV_ related to the same movement. 

### 3.2. Body Stability

Results related to the body stability tests were reported in Table 3. All participants were able to perform the test in EO condition. Conversely, as concerns the test performed with closed eyes, it is worth emphasizing that NK group was not considered in statistical tests since only three subjects of NK succeeded in completing test, while 15 subjects had to rest the contralateral limb during the execution of the test. For this reason, the comparisons among KU, KA, and NK were performed only in the EO, whereas *t*-tests between KU and KA were conducted on the parameters related to EC test and on the RI. 

As regards EO condition, statistical differences were found between KU and NK for the EA (*p* < 0.01), PL (*p* = 0.01) and PL_AP_ (*p* = 0.01), while the only difference between NK and KA was observed for the FREQ_AP_ (*p* < 0.01). No significant differences were found between KU and KA (*p* ranged from 0.10 to 0.91).

By moving to the tests performed with closed eyes, no statistical differences were found between KU and KA (*p* ranged from 0.20 to 0.56). Finally, the Romberg Index (RI) computed for EA of KA was significantly lower in KA than the one computed for KU (*p* = 0.02).

### 3.3. Jumping Ability

**Figure 7:**
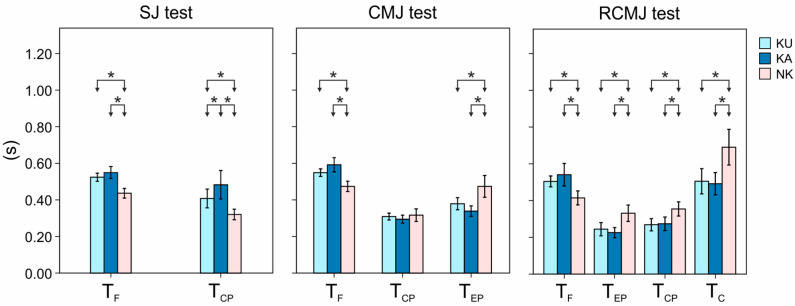
Mean values and standard errors of the parameters related to the jumping tests. From left to right: Squat Jumps (SJ), Countermovement Jumps (CMJ), and Repeated Countermovement Jumps (RCMJ) tests. Parameters: Flight Time (T_F_), Concentric Phase Time (T_CP_), Eccentric Phase Time (T_EP_), and Contact Time (T_C_). KU, KA, and NK stand for kumite, kata, and control group, respectively. * indicates statistical differences.

Mean values and standard errors of the indices related to the SJ, CMJ, and RCMJ tests are reported in Figure 7 with related statistical differences. 

As regards SJ, T_F_ showed significant differences between the NK and karate athletes (*p* always lower than 0.01), with the NK having the lowest average value. Relative to the T_CP_, statistical differences were found among all the examined groups (*p* always lower than 0.01), with the KA showing the highest average value.

By analyzing CMJ, T_F_ and T_EP_ parameters showed statistical differences between the NK and the karatekas (*p* always lower than 0.01); specifically, NK showed the lowest values for T_F_ and the highest for T_EP_. No significant differences were found between the two groups of karatekas. No difference among groups was found for T_CP_ (*p* = 0.32).

By moving to the RCMJ test, statistical differences were found between NK and the karatekas for all the examined parameters (*p* always lower than 0.01). In particular, NK showed lowest average values for T_F_ and the highest ones for T_C_, T_CP_, and T_EP_. No statistical difference was shown between karatekas (*p* ranged from 0.15 to 0.72). 

## 4. Discussion

Joint mobility, body stability, and jumping ability were assessed on sixteen kumite karatekas, eight kata karatekas, and eighteen non-karatekas to: (i) assess the differences between karatekas and non-karatekas to provide guidelines for training programs in beginners; and, (ii) evaluate the differences induced by the karate technique on these kinematic parameters to provide suggestions for coaching programs addressed to elite athletes based on the specific karate technique. 

*Which are the kinematic characteristics that differ karatekas and non-karatekas? Is it possible to provide guidelines for training programs addressed to beginners?*

By discussing the mobility results, the presence of statistical differences between non-karatekas and both karatekas’ groups, with the only exception of the shoulder extension at the preferred velocity, allows affirming that joint mobility and agility is fundamental for the physical preparation of a karateka, as also reported in previous studies [15,44]. More specifically, the greatest differences between karatekas and non-karatekas found for the shoulder and hip joints confirmed the results reported by Podrigalo et al., who assessed as the two mentioned joints are the most involving in karatekas due to the punching and kicking specialization in martial arts [44]. Monopodalic equilibrium test revealed itself as a complex task, and only karatekas were able to perform it in both open and closed eye conditions. This finding suggests that karate training considerably increases body stability [1]. As regards the jumping tests, the differences found between karatekas and non-karatekas for all the examined parameters confirmed as motor ability in jump execution represents one of the physical aspects to train in karatekas. In addition, the differences found for the RCMJ tests can be ascribed to an early occurrence of the fatigue effects in non-karatekas [45]. These findings are in line with the ones reported in Reference [15]. We can speculate that jumping skills and resistance to fatigue are more developed in karatekas due to the specific training program addressed to the jump kicks [46].

Taking into account the above discussed outcomes, we can identify the physical characteristics to be trained in beginners in order to improve their motor performance in karate. Specifically, the differences found for all the examined motor abilities between karatekas and non-karatekas group lead to the conclusion that coaching programs addressed for karate beginners should stress the following aspects: upper and lower limb mobility, movement velocity, body stability, and jumping ability. More specifically, it is clear that the shoulder and the hip represent the most important joints to train since they are the main actors in punching and kicking gestures, which are the main movements performed by a karateka during a competition [46]. This speculation is in line with the outcomes reported by Reference [47], in which authors assessed the training of both the shoulder through throwing tasks and the hip by means of sit and reach tasks, and found that they allow increasing joint mobility in primary school children. Concerning the movement velocity, beginners should pay attention on two aspects: the explosion of the movement but also the control of the gesture; in fact, we have demonstrated that the movement velocity differs for the two techniques. From this perspective, training programs based on sound feedback have revealed themselves to be useful for the beginners [48]. 

In addition, it appears evident as the body stability in monopodalic task represents the weak spot of non-karatekas; thus, interventions focused on the improvement of stability also in single support condition should have explicit attention in beginners training sessions. In fact, it is demonstrated as monopodalic stability strategies are fundamental for providing upper and lower limb gestures with high intensity [49]. Finally, the right trade-off between eccentric and concentric phase, as well as flight and contact time, during jumping has to be carefully trained in order to improve the movement explosion during jump kicks [46]. As a conclusion, we can also suggest to constantly monitor these parameters to evaluate if the planned training programs are causing the desired improvements in the motor abilities. The acquisition of these knowledges can lead to overcome errors in teaching, especially in the initial period of training.

*Which are the effects of karate techniques on joint mobility, body stability and jumping ability? Is it possible to provide guidelines for training programs addressed to elite athletes based on karate specialization?*

The joint angles related to the shoulder extension, hip flexion, and ankle flexion related to the movements performed at the preferred velocity reveal themselves as the mobility indices that are more influenced by the karate technique. As regards the shoulder, the finding can be associated to the gesture required during kumite and kata competition. In fact, unlike the KU, the recall of the upper limb is not foreseen after a punch in the KA. Conversely, the recall of the upper limb, which requires a shoulder extension, represents a typical gesture of the KU after attacking the opponent; in fact, a penalty is assigned during official competition if the kumite athlete hits the opponent without calling the arm back [22]. The outcomes related to the hip flexion confirmed the one reported in Reference [15]. In fact, kumite athletes need a greater hip mobility since they must execute high kicks with an adequate velocity to defeat the opponent; instead, kata athletes can perform more controlled movements. The lower values of mobility that KU showed, with respect to the KA in the ankle joint, can be justified by the different ankle movements required by the two discipline and confirmed the results reported by Reference [20]. During their competitive activities, kumite athletes are often on the forefoot by making small hops in order to optimize the reaction time in case of attack or defense [4]. Such behavior causes an almost constant eccentric-concentric stress on the muscles of the leg, particularly on the triceps surae, leading to an increase of ankle rigidity and, consequently, to a reduction of the range of motion [50,51]. 

As regard the movement velocity at the preferred speed, the lower value of velocity reported for the kata, with the exception of the ankle joint, is directly connected to the specialization required by the technique. In fact, KA athletes perform fine and controlled gestures during their exhibition. This difference was not confirmed in tasks performed with maximum velocity, suggesting that the lower velocity in mobility tasks performed at the preferred velocity for the kata athletes with respect to the kumite ones is effectively due to the technique rather than to less agility of the kata athletes. Nevertheless, the use of the velocity as a useful index should be deeply examined due to the high intra-group variability, which suggests a high heterogeneity also among athletes specialized in the same technique. 

By moving to the body stability, the high values of standard error associated with all the parameters suggest that each karateka adopted a different strategy to complete the task regardless the specific technique. The absence of statistical differences between the two techniques is in contrast with the results reported in Reference [1], in which kata showed greater stability than kumite athletes. However, it should be underlined that the observed better balance skills are related to bipodalic tests. A monopodalic test should be considered more appropriate to assess stability skills of a karateka since athlete is often in a single support position when kicking during the competition. Thus, the absence of statistical difference suggests that both techniques require a high level of body stability. The only parameter useful to differentiate the two karateka groups is the Romberg Index (RI) related to the ellipse area. The lowest value associated with the KA athletes described a better adaptation of KA group in performing balance tests without the aid of the visual apparatus. Thus, it is possible to assess that kata athletes develop stability skills, mainly involving the proprioceptive inputs rather than the visual ones; instead, kumite athletes are skillful in reacting to visual stimuli due to opponent movements both during training and competition. Thus, we can speculate that the KA athletes suffer less from the lack of visual input in balance management [52].

The outcomes obtained from the jump test analyses allow affirming that the two techniques do not lead to different motor performance in jumping due to the general absence of statistical differences between kata and kumite athletes. The only useful parameter seems to be the duration of the concentric phase during squat jump, which KA group showed a longer duration than KU. This result can be ascribed to the starting position of KA athletes characterized by a greater value of the knee angles, leading to deeper squat than the KU athletes. The different starting position can be justified considering two factors. Firstly, it can be linked to the different ankle mobility previously discussed, allowing to reach a lower starting position for the jump for the kata. Secondly, it can be attributed to the different specialization due to the discipline as KA athletes often assume squat positions, similar to SJ, when a push phase is needed from an isometric situation [53].

Considering the above discussed outcomes, we can speculate that a single wearable sensor and the use of optoelectronic bars can allow to provide synthetic indices useful to monitor karate specialization and to focus coaching programs for emphasizing the motor abilities required by the specific karate technique. Focusing on joint mobility, kumite athletes should focus their attention mainly on the shoulder mobility, especially related to the extension movement; instead, kata should stress the training of the ankle mobility. Even though the ankle mobility is shown as one predictive factor, we can speculate that also kumite athletes should take into account the training focused on the ankle mobility; in fact a poor mobility, i.e., joint rigidity, can lead to a worsening of the performance during jumping, as found in the examined jumping tests and also reported in Reference [54]. Furthermore, it has already been demonstrated as an excessive of ankle rigidity can lead to an increase of injuries [55]. From the same perspective, it would be useful the introduction of specific training sessions addressed to increase the ankle mobility during the training programs of kumite athletes, for example, through BHC (Banded Heel Cord) and BCS (Barbell Calf Smash) programs that allow improving ankle mobility with respect to traditional calf stretch technique [56]. In addition, kata training should have specific attention to the control of the movement by avoiding uncontrolled gesture during competition [22].

## 5. Conclusions

Through the aim of quantifying the effects of different karate techniques on motor performance, joint mobility, body stability, and jumping ability were assessed by analyzing twenty-four karateka and eighteen non-karateka healthy subjects. A single inertial sensor and optoelectronic bars data were used to acquired data during the execution of specific tasks. Karatekas showed better motor performance in terms of joint mobility, body stability, and jumping ability than non-karatekas. Mobility outcomes reveal themselves as the most influenced by the specific karate techniques, especially when looking at shoulder and ankle joints. The greater ankle mobility of kata than kumite also leads to a better performance in jumping. Kata athletes are, finally, performing better than kumite athletes when monopodalic stability tests are executed with closed eyes. The findings of this study can be useful for coaching program implementation addressing both beginners and karatekas who want to reach elite level in a specific technique.

## Figures and Tables

**Table 1:** Mean values (standard errors) for θ and $\dot{\theta }$ obtained during the joint mobility test performed at the preferred velocity for all the examined groups. KU, KA, and NK stand for kumite, kata, and non-karatekas group, respectively. Superscripts represent statistical differences, with * indicating difference among all groups.

	Movement	*θ* (°)			$\dot{\theta }$ (°/s)		
		KU	KA	NK	KU	KA	NK
Shoulder	Flexion	179.2 (2.1) ^NK^	172.0 (2.1) ^NK^	160.1 (1.8) ^KA,KU^	278.4 (24.6) ^NK,KA^	143.4 (23.2) ^KU^	199.1 (8.4) ^KU^
	Extension	82.1 (2.3) ^NK,KA^	68.1 (2.8) ^KU^	66.3 (1.8) ^KU^	178.8 (20.3) ^NK,KA^	90.5 (19.1) ^KU^	105.0 (6.5) ^KU^
	Abduction	172.6 (1.7) ^NK^	171.4 (2.3) ^NK^	155.0 (2.2) ^KA,KU^	306.1 (26.8) ^NK,KA^	148.7 (30.9) ^KU^	215.7 (10.3) ^KU^
	Extra-Rotation	108.1 (1.9) ^NK^	105.6 (2.4) ^NK^	89.4 (1.7) ^KA,KU^	229.6 (22.7) ^NK,KA^	115.7 (20.1) ^KU^	166.8 (9.9) ^KU^
Hip	Flexion	122.4 (2.2) *	112.6 (3.3) *	82.1 (1.8) *	213.4 (15.0) ^NK,KA^	119.5 (20.1) ^KU^	100.7 (4.9) ^KU^
	Split	78.0 (1.5) ^NK^	76.5 (2.2) ^NK^	49.5 (1.7) ^KA,KU^	155.9 (8.6) *	105.0 (13.3) *	37.7 (2.8) *
	Sit and Reach	37.3 (2.5) ^NK^	36.7 (2.6) ^NK^	19.4 (2.1) ^KA,KU^	59.8 (6.9) ^NK^	51.0 (6.6) ^NK^	16.5 (2.4) ^KA,KU^
Ankle	Flexion	71.5 (1.1) *	80.5 (1.5) *	57.9 (2.0) *	96.5 (11.2)	80.1 (10.4)	121.3 (11.0)
	Overhead squat	29.2 (1.3) ^KA^	37.8 (1.3) ^KU,NK^	28.8 (1.0) ^KA^	32.6 (4.1)	34.0 (4.7)	24.5 (2.1)

**Table 2:** Mean values (standard errors) for θ_MV_ and $\dot{\theta }$_MV_ obtained during the joint mobility test performed at the maximum possible velocity for all the examined groups. KU, KA, and NK stand for kumite, kata, and non-karatekas group, respectively. Superscripts represent statistical differences, with * indicating difference among all groups.

	Movement	$\theta_{MV}$ (°)			${\dot{\theta }}_{MV}$ (°/s)		
		KU	KA	NK	KU	KA	NK
Shoulder	Flexion	192.0 (1.7) ^NK^	190.2 (2.2) ^NK^	171.0 (1.8) ^KA,KU^	550.4 (18.9) ^NK^	491.1 (21.6) ^NK^	407.4 (12.9) ^KA,KU^
	Extension	100.9 (2.9) ^NK^	94.7 (2.4) ^NK^	80.8 (1.7) ^KA,KU^	378.8 (14.5) ^NK^	346.1 (21.6) ^NK^	234.2 (10.7) ^KA,KU^
	Abduction	179.2 (1.7) ^NK^	180.4 (1.8) ^NK^	162.0 (2.0) ^KA,KU^	522.3 (14.4) ^NK^	474.1 (23.9) ^NK^	392.9 (9.3) ^KA,KU^
	Extra-Rotation	117.6 (1.7) ^NK^	120.6 (2.7) ^NK^	96.2 (2.3) ^KA,KU^	529.4 (15.9) *	448.2 (17.8) *	354.7 (16.0) *
Hip	Flexion	139.5 (1.9) ^NK^	137.0 (3.2) ^NK^	94.7 (2.7) ^KA,KU^	331.5 (10.8) ^NK^	295.4 (14.1) ^NK^	190.3 (6.8) ^KA,KU^

**Table 3:** Mean values (standard errors) for the body stability tests for all the examined groups. KU, KA, and NK stand for kumite, kata, and non-karatekas group, respectively. Superscripts represent statistical differences. EO and EC indicate test performed with open and closed eyes, respectively. * Romberg Index (RI) is adimensional for each parameter.

	EO			EC		Romberg Index *	
	KU	KA	NK	KU	KA	KU	KA
Path Length (cm)	48.9 (3.4) ^NK^	65.8 (8.7)	68.8 (4.7) ^KU^	119.0 (12.3)	120.9 (15.0)	2.4 (0.3)	1.8 (0.2)
Path Length AP (cm)	33.8 (2.7) ^NK^	43.8 (5.4)	46.0 (3.1) ^KU^	76.6 (9.1)	72.6 (8.1)	2.3 (0.3)	1.7 (0.2)
Path Length ML (cm)	28.3 (1.9)	39.4 (6.3)	42.6 (3.4)	74.6 (6.7)	79.7 (11.9)	2.7 (0.3)	2.1 (0.3)
Ellipse Area (cm^2^)	36.4 (7.4) ^NK^	55.4 (14.7)	92.7 (13.2) ^KU^	179.0 (38.3)	204.9 (47.0)	9.1 (2.4) ^KA^	3.9 (0.7) ^KU^
Frequency (Hz)	0.19 (0.01)	0.19 (0.02)	0.13 (0.05)	0.20 (0.01)	0.18 (0.02)	1.20 (0.10)	1.06 (0.08)
Frequency AP (Hz)	0.22 (0.02)	0.26 (0.03) ^NK^	0.16 (0.01) ^KA^	0.22 (0.01)	0.22 (0.03)	1.23 (0.12)	1.12 (0.16)
Frequency ML (Hz)	0.23 (0.02)	0.24 (0.05)	0.16 (0.01)	0.26 (0.18)	0.22 (0.04)	1.32 (0.15)	1.17 (0.12)
